# Replacement of grains with soybean hulls ameliorates SARA-induced impairment of the colonic epithelium barrier function of goats

**DOI:** 10.1186/s12917-018-1705-8

**Published:** 2018-12-03

**Authors:** Kai Zhang, Yuanlu Tu, Lipeng Gao, Meijuan Meng, Yunfeng Bai

**Affiliations:** 1 Circular Agriculture Research Center, Jiangsu Academy of Agricultural Sciences, Nanjing, China; 2 Key Laboratory of Crop and Livestock Integrated Farming, Ministry of Agriculture, Nanjing, China

**Keywords:** Goat, SARA, Colonic epithelium, Soybean hulls, Tight junction proteins

## Abstract

**Background:**

The effect of soybean hull feeding on the disruption of colonic epithelium barrier function was investigated in goats fed a high-concentrate diet. Twenty-one Boer goats (live weight, 32.57 ± 2.26 kg; age, 1 year) were randomly divided into three groups: low-concentrate diet (LC), high-concentrate diet (HC), and high-concentrate diet with soybean hulls (SH).

**Results:**

We found that the rumen fluid in the LC and SH group shown a higher pH value compared with the HC group. The mRNA and protein expression levels of extracellular regulated protein kinase (ERK), c-Jun N-terminal kinase (JNK), and p38 mitogen-activated protein kinase (MAPK) in the colonic epithelium were significantly decreased in the SH group than in the HC group. Moreover, in goats fed the HC diet, SH treatment promoted gene expression and protein abundance of claudin-1, claudin-4, occludin, and ZO-1 in the colonic epithelium. Additionally, the injury to the colonic epithelium barrier caused by the HC diet was reversed by SH treatment.

**Conclusions:**

Our results indicated that supplemental SH feeding reverses the damage to colonic epithelium tight junctions by inhibiting the MAPK signalling pathway and has a protective effect on the colonic epithelium during SARA.

## Background

Ruminants are always fed a large amount of cereals in their diet to meet the nutritional requirements for energy for rapid weight gain or high milk yields. Although grains are beneficial in the short term, the risk of a metabolic disorder and systemic disease termed subacute rumen acidosis (SARA) increases after long-term feeding [1]. A ruminal pH between 5.6 and 5.8 not less than 3 h per day is the most widely accepted parameter in the diagnosis of SARA [2, 3]. This low ruminal pH perturbs the balance of rumen microbial populations, which is directly related to the release of lipopolysaccharide (LPS) endotoxin from lysed bacteria in the gastrointestinal tract and to damage to the gastrointestinal epithelium [4, 5]. Free LPS can be translocated from this dietary-induced damage to the gut mucosa, causing a systemic inflammatory response [6–8].

The colonic epithelium is a polarized monolayer of columnar epithelial cells that form a semipermeable paracellular diffusion barrier for nutrient absorption and metabolism, prohibiting the transportation of microbes and toxins under the normal physiological state [9]. However, the barrier properties of the gastrointestinal epithelium can be damaged by low pH and hyperosmolarity when SARA occurs, resulting in increased translocation of LPS into circulation [10, 11]. Epithelial cells form selective barriers with an elaborate network of intercellular protein complexes tight junctions (TJs). TJs regulate integral mechanisms of epithelial morphogenesis, which are crucial for proper function and formation of epithelial barriers. The transmembrane proteins claudins and occludin and cytoplasmic plaque composed of the proteins zonula occludins (ZO)-1, − 2, and − 3 are by far the most important for TJs [12–14].

The abnormal expression of TJ protein can lead to the impairment of the colon barrier [15]. The expression of occludin is correlated with various barrier properties [16, 17], and overexpression of occludin leads to changes in the gate and fence function of TJs in mammalian epithelial cells [18, 19]. Additionally, the occludin loop peptides can lead to the disappearance of TJs [20]. Although tight junctions exhibit normal barrier properties in occludin-knockout mice, complex abnormalities are observed, with postnatal growth retardation, the absence of fertility, the thinning of compact bone, chronic inflammation and hyperplasia [21, 22]. Claudins are identified as key molecules in the barrier function of TJs [23]. Overexpression of claudin-1 and -2 in fibroblasts can reconstitute tight junction strands [24], and multiple lines of evidence suggest that overexpression or knockout of claudins can ameliorate junctional ion permeability and barrier function [25, 26]. ZO-1, the first tight junction protein identified, interacts with multiple other junctional components, including claudins and occludin [27]. A previous study demonstrated that TJ barrier formation disintegrated because of the depletion of ZO-1 in cultured epithelial cells [28]. Moreover, claudin and occludin cannot constitute a normal TJ structure in ZO-depleted cells. MAPK signalling pathways regulate the expression of TJ proteins. We found that the activity of the MAPK signalling pathway can disrupt epithelial barrier function together with a decrease transcriptional level in claudin-1, occludin, and ZO-1 [29, 30].

Recently, replacing grain with low-starch, nonforage fibre has become a potential alternative to prevent SARA occurrence. A previous study demonstrated that partial substitution of starch with nonforage fibre sources, such as soybean hulls, positively affected milk production and composition in lactating dairy cows. Moreover, this substitution can increase feed efficiency and decrease diet feed costs [31]. Our previous studies have indicated that soybean hulls are traditionally fed as a protein supplement to improve the low rumen pH induced by SARA and do not affect the performance of goats. However, little is known about whether soybean hulls can prevent colonic epithelial barrier dysfunction when grain-induced SARA occurs. Therefore, the present study was conducted to confirm that soybean hulls increase the expression of TJs through modification and signalling pathway regulation, to provide a new treatment option for impaired barrier function.

## Results

### Rumen pH value analysis

**Fig. 1:**
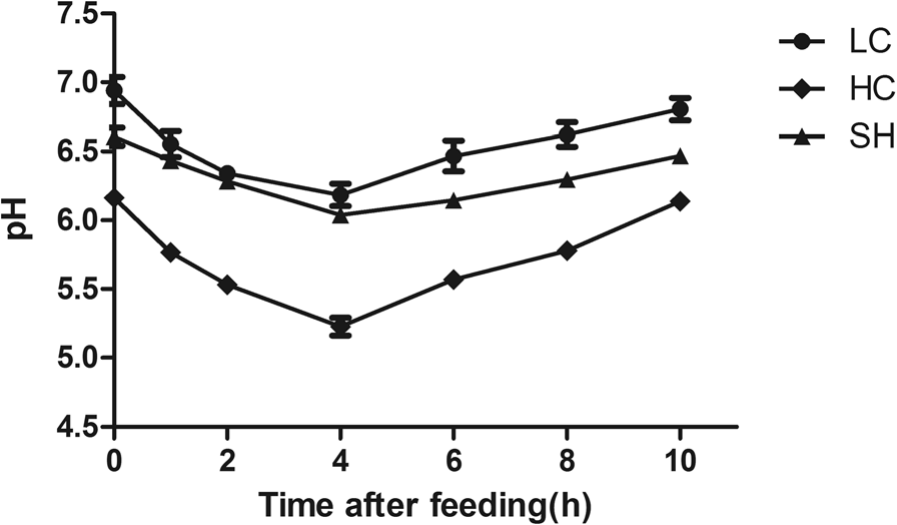
Rumen pH value of different groups at different time points after feeding. The pH was markedly decreased in the HC group. With supplemental feeding with soybean hulls (SH), the pH changed significantly compared with that in the HC group. The error bars indicate the standard error of the mean. All of the data are shown as the mean ± SD. Differences between two groups were considered significant when *P* < 0.05

The average rumen pH value in the LC and SH group was markedly higher than that in the HC group (Fig. 1, *P* < 0.05). A reduced pH value between 5.6 and 5.8 was approximately 4 h/day in the HC group of animals. The rumen pH value in the LC and SH groups was similar.

### pH and LPS content in the colon and LPS and cytokines in plasma

Colonic pH value decreased from 6.90 for the LC group to 5.98 for the HC group (Table 1, *P* < 0.05). There was no significant difference in the colonic pH between the LC and SH groups.

**Table 1:** LPS and primary pro-inflammatory cytokines content of different groups

Item	LC	HC	SH
pH in colon	6.90^a^±0.34	5.98^b^±0.42	6.73^a^±0.28
LPS in colon, kEU/mL	27.64^b^±2.96	56.3^a^±3.26	28.3^b^±3.83
LPS in plasma, EU/mL	0.16^b^±0.02	0.96^a^±0.35	0.23^b^±0.06
IL-1β in plasma, ng/mL	0.162^b^±0.013	0.23^a^±0.018	0.155^b^±0.016
IL-6 in plasma, pg/mL	15.67^b^±1.17	72.2^a^±0.68	12.2^b^±0.41
TNF-α in plasma, fmol/mL	76.73^c^±5.32	570.36^a^±24.29	354.15^b^±51.42

Different characters (a, b and c) show significant difference among diets (*p* < 0.05)

Colonic content and plasma LPS concentration in the LC and SH group was significantly lower than that in the HC group (*P* < 0.01), while the concentration of LPS in the LC and SH groups was similar, as shown in Table 1.

IL-1β, IL-6, and TNF-α concentrations in the HC group were significantly increased (*P* < 0.01, Table 1) compared with those in the LC group, while the concentrations of IL-1β and IL-6 had no significant difference between the SH group and LC group. The level of TNF-α was significantly higher in the SH group than in the LC group (*P* < 0.05, Table 1).

### Morphological analysis of the colonic epithelium

**Fig. 2:**
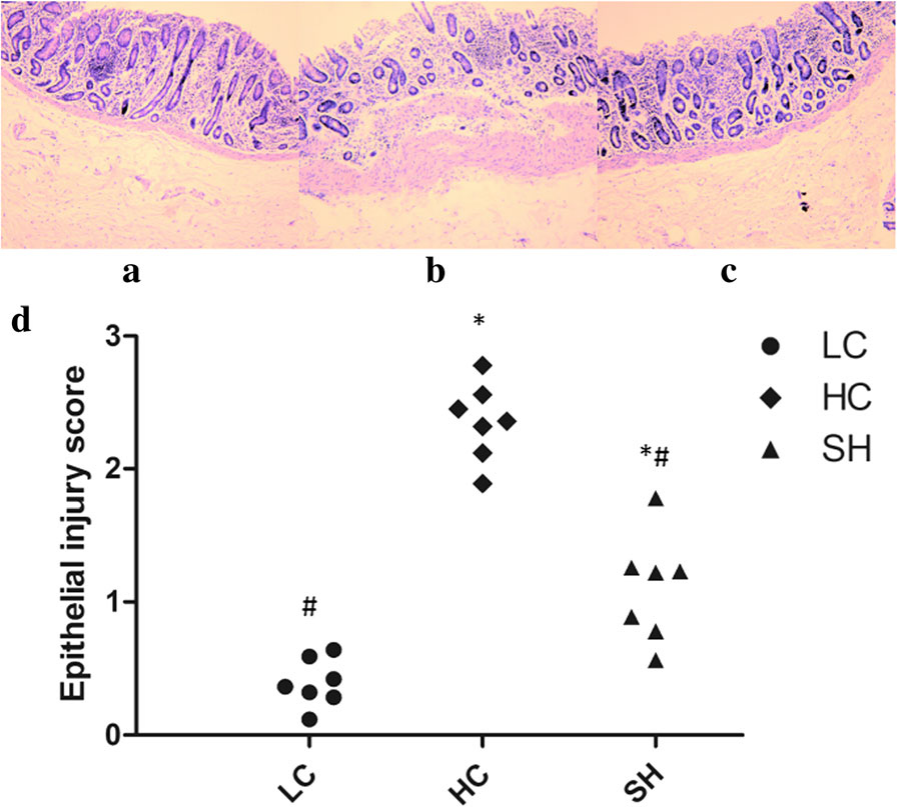
Histological alteration of the colonic epithelium of goats from the different groups. Representative photomicrographs with haematoxylin and eosin staining. The colonic epithelium of the LC group was intact and showed no disruption (**a**), while the stratum corneum structure of the epithelium was severely damaged in the HC group (**b**). Slight damage of the epithelium was observed in the SH group (**c**). Epithelial injury scores are shown in **d**. * indicates *P* < 0.05 and ** indicates *P* < 0.01 compared with the LC; # indicates *P* < 0.05 compared with the HC. A statistically significant difference has a *P* value < 0.05

The epithelial injury scores confirmed the results of the colonic epithelium histological analysis, as shown in Fig. 2. The epithelial surface of colonic epithelium was sloughed in the HC group, while it in the LC and SH group covered by mucus and remained intact. Although the inflammatory cells in the colom serosal muscle layer of the SH group were more than the LC group, it was much less than the HC group. A high-concentrate diet with soybean hulls feeding had a marked decrease macroscopic damage score compared with the HC group (*P* < 0.05).

### Relative expression of genes related to barrier function in the colonic epithelium

**Fig. 3:**
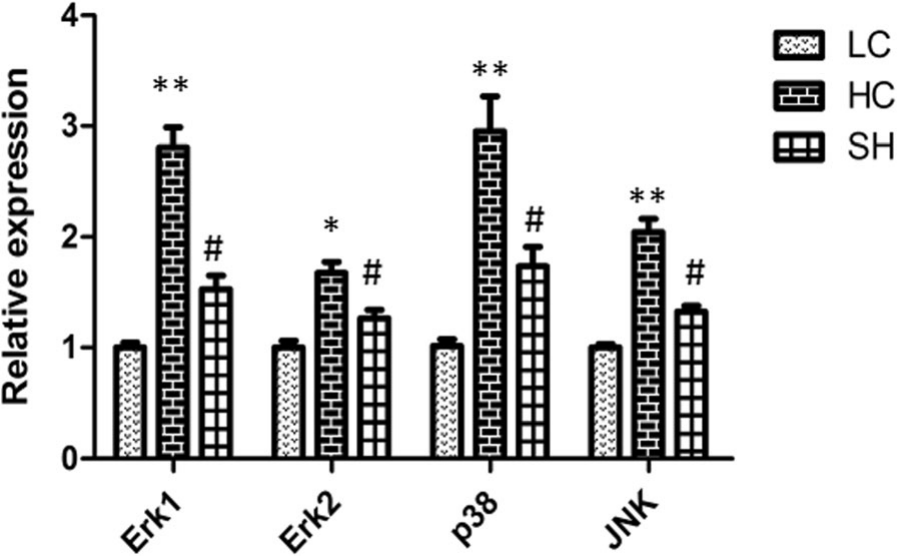
The expression of genes related to the MAPK signalling pathway in the colonic epithelium of goats in the LC, HC, and SH groups. The expression of Erk1, Erk2, p38 MAPK and JNK significantly increased in the HC group compared with that in the LC group, while there was no significant difference between the SH and LC groups. The results are expressed as fold changes relative to the LC group (mean ± SEM). * indicates *P* < 0.05 and **indicates *P* < 0.01 compared with the LC; # indicates *P* < 0.05 compared with the HC. A statistically significant difference has a *P* value < 0.05

**Fig. 4:**
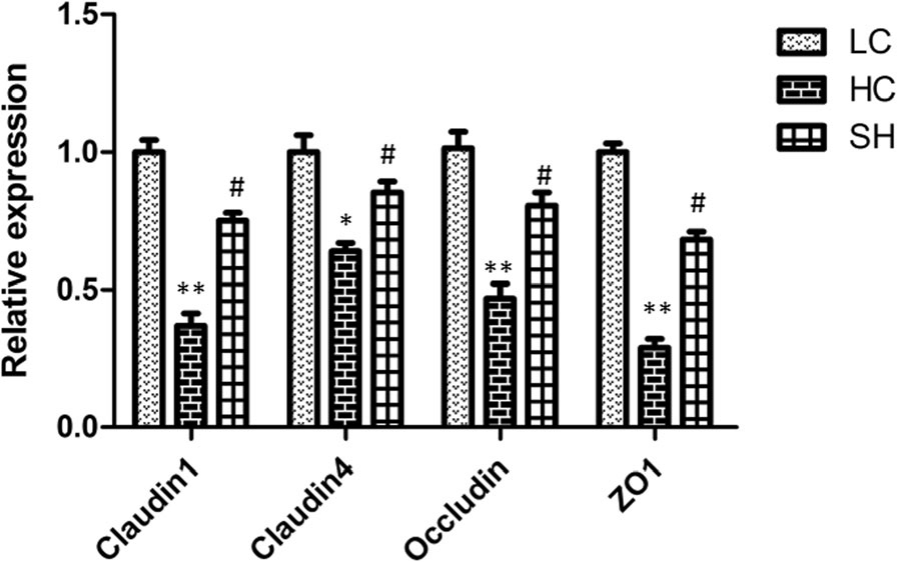
The expression of genes related to tight junction proteins in the colonic epithelium of goats in the LC, HC, and SH groups. The colonic epithelium of the goat fed the HC diet had a significant decline in the mRNA expression of claudin-1, claudin-4, occludin, and ZO-1 compared with that in the LC group, while no significant change between the SH and LC groups was observed. The results are expressed as fold changes relative to those in the LC group (mean ± SEM). * indicates *P* < 0.05 and **indicates *P* < 0.01 compared with the LC; # indicates *P* < 0.05 compared with the HC. A statistically significant difference has a *P* value < 0.05

Several pivotal genes were chosen to determine the colonic epithelium barrier function of the goats fed different diets, and the results are shown in Figs. 3 and 4. The mRNA relative expression of ERK1, p38 and JNK, increased in the HC group compared with that in the LC group (*P* < 0.01), while these in the LC and SH groups was similar. The expression of ERK2 was also increased markedly in the HC group (*P* < 0.05).

The mRNA expression of claudin-1, claudin-4, occludin, and ZO-1 was also detected by RT-qPCR. The mRNA expression of these genes in the colonic epithelium of goats fed the HC diet declined significantly compared with the LC group, while the expression in the LC and SH groups was similar.

### Phosphorylation levels of ERK, pERK, JNK, pJNK, p38, and pp38 in the colonic epithelium

**Fig. 5:**
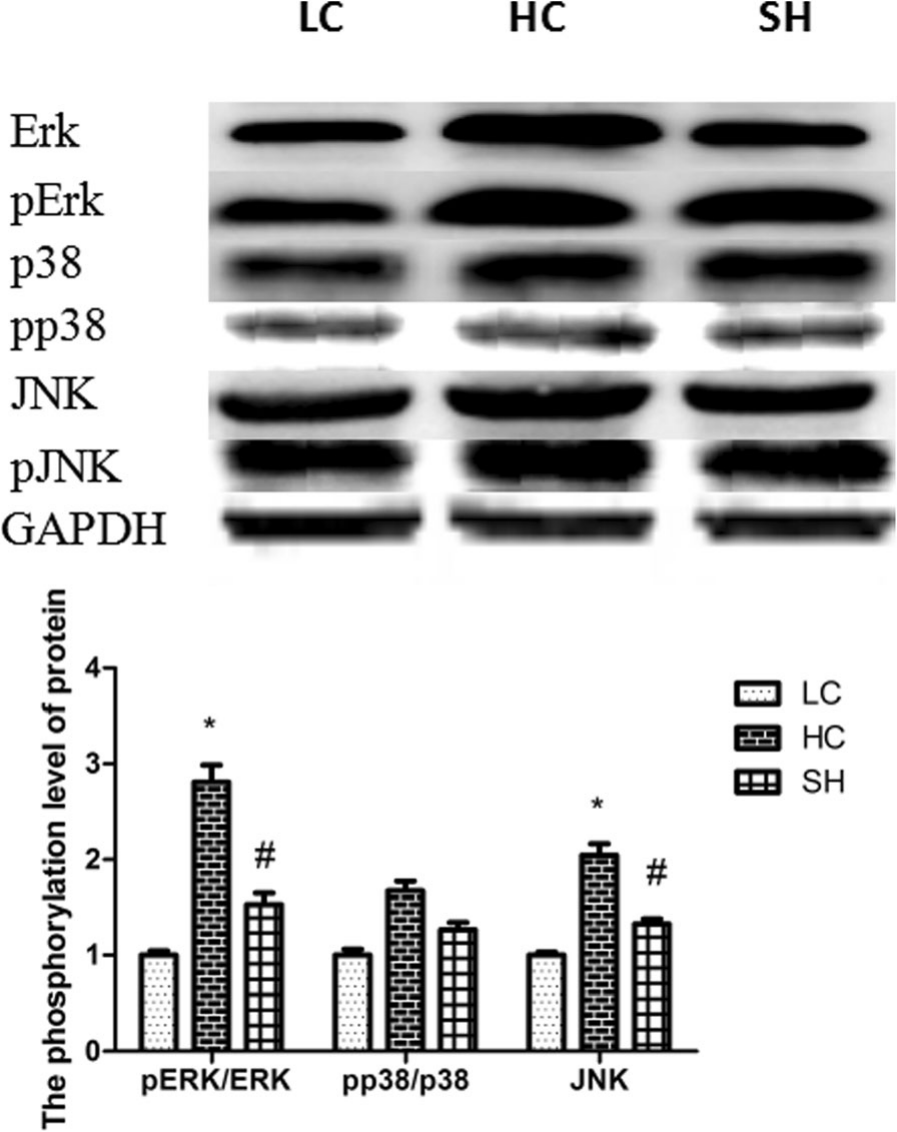
The phosphorylation levels of Erk, JNK and p38 MAPK in the colonic epithelium of goats in the LC, HC, and SH groups. Erk, JNK and p38 MAPK phosphorylation levels in the HC group were significantly elevated compared with those in the LC group, while the levels in the SH group were not elevated. The results are expressed as fold changes relative to those in the LC group (mean ± SEM). * indicates *P* < 0.05 and ** indicates *P* < 0.01 vs the LC; # indicates *P* < 0.05 compared with the HC. A statistically significant difference has a *P* value < 0.05

The phosphorylation levels of ERK, p38 and JNK were determined by Western blot analysis (Fig. 5). ERK and JNK phosphorylation levels in the HC group were significantly elevated compared with those in the LC group, while the levels in the SH group were not elevated. There was no significant difference in the phosphorylation levels of p38 among the treatments.

### Protein levels of claudin-1, occludin, and ZO-1 in the colonic epithelium

**Fig. 6:**
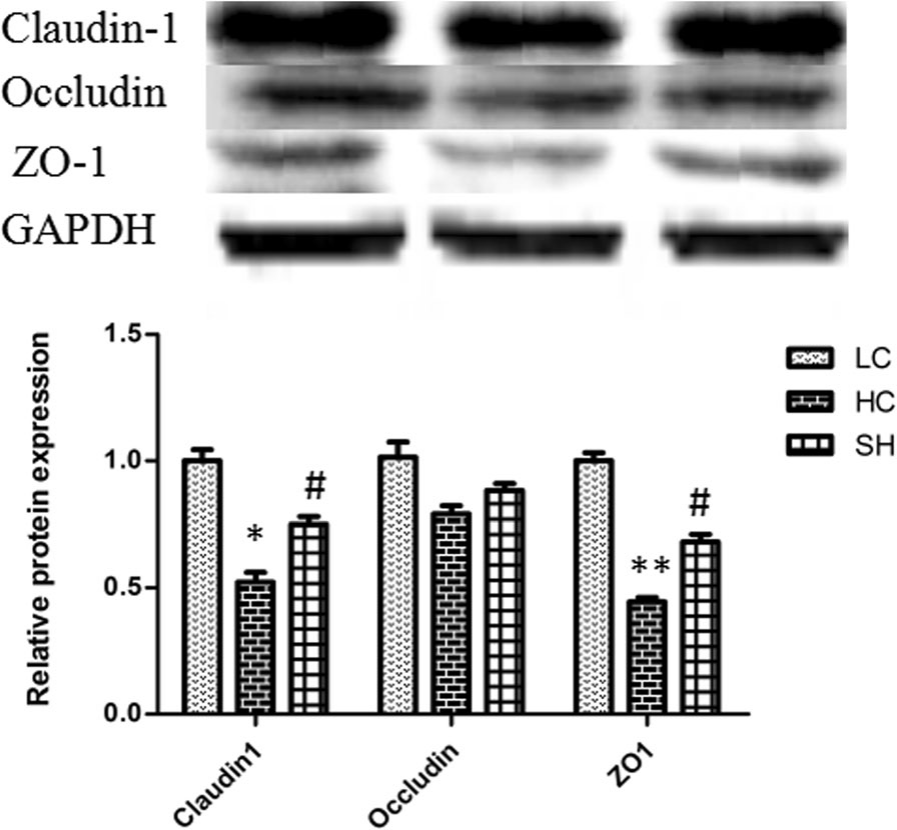
Protein abundance of claudin-1, occludin, and ZO-1 in the colonic epithelium of goats in the LC, HC, and SH groups. The abundance of claudin-1 and ZO-1 proteins in the HC group significantly decreased compared with that in the LC group, while the levels in the SH group were not significantly altered. The results are expressed as fold changes relative to those in the LC group (mean ± SEM). * indicates *P* < 0.05 and ** indicates *P* < 0.01 vs the LC; # indicates *P* < 0.05 compared with the HC. A statistically significant difference has a *P* value < 0.05

Western blotting of claudin-1 and ZO-1 showed significantly lower protein levels in the HC group than in the LC group, whereas the levels in the SH and LC groups were similar (Fig. 6). The protein expression of occludin was not significantly different among the three groups.

## Discussion

Feeding an HC diet to ruminants can not only promote microbial protein synthesis in the rumen and increase growth and milk production but also negatively affect rumen function because of the rapid accumulation of volatile fatty acids (VFA), which leads to decreased ruminal pH [32, 33]. Goats fed the HC diet for 12 weeks exhibited a low rumen pH between 5.6 and 5.8 for more than 3 h, which met the current definition of SARA, indicating that SARA was successfully induced by the HC diet in the present study. We found that supplementing the HC diet with soybean hulls in the SH group led to a higher rumen pH than that in the HC group. Soybean hulls contain a high concentration of digestible neutral detergent fibre, which can increase chewing time and saliva secretion [31], possibly explaining how the SH diet increased the rumen pH. A low ruminal pH leads to gastrointestinal epithelial barrier dysfunction and translocation of LPS, increasing the risk of system inflammatory response [34, 35]. Based on our results, concentrations of LPS in the colon content and plasma of goats in the HC group were higher than those in the LC and SH groups.

Feeding the animals the HC diet could interrupt the barrier function of the colonic epithelium in our study. The expression of claudin-1, claudin-4, occludin, and ZO-1 declined significantly in the HC group, providing evidence that HC feeding can modulate the expression of TJ proteins and disrupt the barrier function. With the addition of SH, the expression of TJs was upregulated to a similar level to the LC group. Claudin-1, claudin-4, and ZO-1 protein abundance in the colonic epithelium of goats in the HC group was downregulated compared with that in goats treated with LC and SH. We also found that SH diet feeding ameliorated the damage to the colonic epithelial barrier induced by HC diet feeding through histological alterations.

The MAPK signalling pathway is widely accepted to play a key role in regulating TJ protein formation [36]. JNK is essential in regulating TJ expression and epithelial barrier function. Inhibition of JNK activity in murine mammary epithelial cells can modulate of claudins expression and increase epithelial barrier function [37]. The expression of TJs can be depleted by various stimuli, such as the JNK activator anisomycin and the pro-inflammatory cytokines in human pancreatic cancer cells [38]. Proinflammatory cytokine treatment can induce the expression of TJ proteins, whereas the change was reversible by a JNK inhibitor. Some studies have indicated that HC diet feeding could enhance immune gene expression and activate the JNK signalling pathway in the liver of dairy cow [39]. These findings suggest that the concentration of pro-inflammatory cytokines and the phosphorylation level of the JNK pathway could induce disruption of the epithelial barrier. In the current study, the production of IL-1β, TNF, and IL-6 in the HC group was significantly higher than that in the LC group. The protein expression of JNK and pJNK increased, consistent with the mRNA expression of the corresponding gene by HC feeding. With SH administration, the indicators remained at levels similar to those observed in the LC group. The overexpression of the junctional membrane protein occludin can suppress ERK/MAP kinase activation [40]. In our study, the mRNA expression of ERK1 and ERK2 in goats induced by HC diet feeding declined after SH treatment, and SH treatment also decreased the phosphorylation levels of ERK. The expression of claudin-1 and -2 can be regulated by the p38 activator in vitro [41]. Decreasing expression of claudin-1 in regenerating rat liver has been reported by the p38 MAP kinase inhibitor [42], and p38 MAP kinase and Akt can inhibit the expression of claudin-4 in hepatic cell lines [43, 44]. Thus, the p38 MAP kinase pathway plays an important role in TJ formation in vivo and in vitro. SH treatment improved the downregulated expression of p38 induced by the HC diet in our study, which is consistent with the increased expression of claudin-1 and claudin-4 in our study. Hence, these lines of evidence collectively prove that the expression of TJs can be regulated by the MAPK signalling pathway in the colonic epithelium of goats.

## Conclusions

According to our results, HC diet feeding for 12 weeks severely compromised the colonic epithelium and impaired epithelial barrier function. These adverse effects were attenuated after SH treatment through the phosphorylation of the MAPK signalling pathway. Thus, a new option can be applied to remedy a series of systemic inflammatory responses induced by HC diet feeding.

## Methods

### Animals, diet, and experimental design

Twenty-one healthy, 1-year-old male Boer goats with an average body weight of 32.57 ± 2.26 kg, which were purchased from the Luhe Experimental Farm of Jiangsu Academy of Agricultural Sciences, were randomly divided into three groups (*n* = 7): low-concentrate diet (LC; 70% forage and 30% concentrate), high-concentrate diet (HC; 30% forage and 70% concentrate), and high-concentrate diet with soybean hulls (SH; 20% soybean hulls instead of grain). The diet nutritional composition are shown in Table 2. The goats were fed these diets at 8:00 a.m. and 6:00 p.m. and provided fresh water for 12 weeks.

**Table 2:** Ingredients and nutrient composition of experimental diets

Ingredient,% DM	Percentage (%)of ingredients in different diets (air dry matter)		
	LC	HC	SH
Maize	12	40	21.78
Soybean meal	11.92	6.36	5.30
Wheat bran	1.00	8.98	8.57
Wheat	2.09	12.00	11.83
Soybean hull	0.00	0.00	20.00
Straw	70	30	30
Calcium hydphosphate	1.40	0.55	0.80
Limestone	0.59	1.10	0.73
Salt	0.50	0.50	0.50
Premix^a^	0.50	0.50	0.50
Total	100	100	100
Nutrient composition			
DE, MJ/kg	8.65	11.43	10.74
CP %	11.77	11.76	11.77
NDF %	44.81	26.53	36.26
ADF %	24.63	13.45	22.23
Ash %	10.26	8.13	8.52

^a^The premix consisted of the following ingredients per kg of diet: 6.60 × 104 IU of vitamin A, 8.00 × 105 IU of vitamin D3, 1.49 × 103 of vitamin E, 35.2 mg of Cu, 120 mg of Fe, 115 mg of Zn, 80 mg of Mn, 0.35 mg of Co, and 19.5 mg of Se

### Sample collection and analysis

The rumen pH values were measured on the last day of weeks 10, 11, and 12 through a cannula at 0, 1, 2, 4, 6, 8, and 10 h after feeding by a basic pH meter.

Blood samples were collected via the lacteal vein into 5-mL vacuum blood collection tubes. The blood samples were centrifuged at 1500×g for 15 min, and the plasma was collected and stored at − 20 °C for the determination of LPS, IL-1β, IL-6, and TNF-α.

The goats were slaughtered according to the law of Jiangsu Provincial People’s Government, China, and the study was approved by the guidelines of Jiangsu Province Animal Regulations (Government Decree No. 45) and the Committee on the Ethics of Animal Experiments of Jiangsu Academy of Agricultural Sciences. The pH values of colonic digesta samples were immediately measured. Colons were removed and washed with PBS. Colonic tissue sample was selected from the same part of the colon aseptically and divided into two parts. One part was cut into 1 cm × 1 cm small pieces and infiltrated in 4% paraformaldehyde solution, and the other part was transferred into liquid nitrogen and then kept at − 80 °C for later detection.

### Determination of LPS content in colon and plasma primary pro-inflammatory cytokines

LPS concentration in the colon content and plasma was determined with Chromogenic Endpoint Limulus Amebocyte Lysate Assay Kits (Chinese Horseshoe Crab Reagent Manufactory Co., Ltd., Xiamen, China). The detection range of LPS concentrations in colon and plasma were 0.1–1 endotoxin units (EU)/mL and 0.01–0.1 EU/mL, respectively. IL-1β, IL-6 and TNF-α concentrations in the plasma were determined by radioimmunoassay radioimmunoassay kits C09DJB, C12DJB and C06PJB purchased from the Beijing North Institute of Biological Technology with a detection limit of 0.1–8.1 ng/mL, 50–4000 pg/mL and 9–590 fmol/mL.

### Histological analysis

Fixed tissues were embedded in paraffin after immersing in a 4% paraformaldehyde solution for 72 h and dehydrating by ethanol. Five micrometer sections were cut form the paraffin on a microtome, mounted on slides, and stained with haematoxylin and eosin. The images were recorded by light microscope and high-resolution digital camera. Histological damage was assessed with a scoring epithelial injury criteria (graded 0 to 3) which has been described previously [45].

### Quantitative real-time PCR

100 mg colonic tissue samples were selected for RNA extraction using RNAiso Plus (TaKaRa, Otsu, Japan). The A260/A230 and A260/A280 ratio of RNA samples were measured by spectrophotometer (Thermo Fisher Scientific Inc., Waltham, MA, United States). Then, RNA was reverse transcribed to the first-strand cDNA by PrimeScript RT kit (Cat. RR036A; TaKaRa). Primers were designed and synthesized in Shanghai Sangon Biotech Co., Ltd. and are listed in Table 3. Quantitative real-time PCR was performed using a SYBR Premix EX Taq Kit (Cat. DRR420A; TaKaRa) via an ABI 7300 system (Applied Biosystems, Foster City, CA, USA) according to the manufacturer’s protocol. Glyceraldehyde-3-phosphate dehydrogenase (GAPDH) was used as the internal reference and the data was analyzed by 2^-ΔΔCt^ method.

**Table 3:** The primer sequences of target and internal reference genes used in qRT-PCR

Gene	Forward primer	Reverse primer	PCR products (bp)
Erk1	CTCAGCTTACGACCATGTGC	TCAGGTCCTGCACGATGTAG	203
Erk2	CTCAGCAACGACCACATCTG	CCAGGCCAAAGTCACAGATC	151
p38	ACAACATCGTCAAGTGCCAG	CACGTAGCCAGTCATCTCCT	209
JNK	TCAGTCAGTTGAGCACCAGT	ACTTATGCCTGCTCTGCTCA	229
Claudin-1	CACCCTTGGCATGAAGTGTA	AGCCAATGAAGAGAGCCTGA	216
Claudin-4	AAGGTGTACGACTCGCTGCT	GACGTTGTTAGCCGTCCAG	238
Occludin	GTTCGACCAATGCTCTCTCAG	CAGCTCCCATTAAGGTTCCA	200
ZO-1	CGACCAGATCCTCAGGGTAA	AATCACCCACATCGGATTCT	163
GAPDH	GGGTCATCATCTCTGCACCT	GGTCATAAGTCCCTCCACGA	180

### Western blot analysis

Approximately100 mg of frozen grated colonic tissue was homogenized in 1 mL ice-cold RIPA protein isolation buffer (Cat. No. SN338; Sunshine Biotechnology Co., Nanjing, China) for the total protein extraction. The protein concentration was measured via the BCA Protein Assay kit (No. 23225, Thermo Fisher, USA). 50 μg of total protein was loaded onto 12% sodium dodecyl sulfate-polyacrylamide gel electrophoresis (SDS-PAGE) and transferred to a nitrocellulose membrane (Bio Trace; Pall Co., Port Washington, NY). The membranes were incubated in the appropriate primary antibodies: Jun N-terminal kinase (JNK) (No. 9252S; Cell Signaling Technology, Danvers, MA), p-JNK (No. 9255S; Cell Signaling Technology), p38 (No. 8690S; Cell Signaling Technology), p-p38 (No. 4511S; Cell Signaling Technology), Erk1/2 (No. 4695S; Cell Signaling Technology), p-Erk1/2 (No. 4370S; Cell Signaling Technology), PKC- (No. 2056S; Cell Signaling Technology), occludin (No. ab167161; Abcam, Cambridge, United Kingdom), claudin-1 (No. ab15098; Abcam), and ZO-1 (No. ab214228; Abcam), then washed and incubated in corresponding horseradish peroxidase (HRP)-conjugated secondary antibodies. Finally, the membranes were washed and visualized using an enhanced chemiluminescence (ECL) Kit (Pierce, Rockford, IL). The signals were recorded using an Bio-Rad imaging system (Bio-Rad, Hercules, CA), and the results were analyzed using Quantity One software (Bio-Rad).

### Statistical analysis

The results are expressed as the mean ± standard error of the mean (SEM). All data were evaluated with an unpaired Student’s t-test or one-way ANOVA with the SPSS 21.0 statistical software package. A *P* value < 0.05 was considered to indicate a significant difference.

## Abbreviations

ERK: Extracellular regulated protein kinases
HC: High-concentrate diet
JNK: c-Jun N-terminal kinase
LC: Low-concentrate diet
LPS: Lipopolysaccharide
MAPK: Mitogen-activated protein kinase
SARA: Subacute ruminal acidosis
SH: Soybean hulls
TJ: Tight junction
TNF-α: Tumour necrosis factor α
